# Fluorescent imaging of superficial head and neck squamous cell carcinoma using a γ-glutamyltranspeptidase-activated targeting agent: a pilot study

**DOI:** 10.1186/s12885-016-2421-z

**Published:** 2016-07-07

**Authors:** Takeshi Mizushima, Shunsuke Ohnishi, Yuichi Shimizu, Yutaka Hatanaka, Kanako C. Hatanaka, Hidetaka Hosono, Yoshimasa Kubota, Mitsuteru Natsuizaka, Mako Kamiya, Shouko Ono, Akihiro Homma, Mototsugu Kato, Naoya Sakamoto, Yasuteru Urano

**Affiliations:** Department of Gastroenterology and Hepatology, Hokkaido University Graduate School of Medicine, N15, W7, Kita-ku, Sapporo, 060-8638 Japan; Department of Surgical Pathology, Hokkaido University Hospital, N14, W5, Kita-ku, Sapporo, 060-8648 Japan; Laboratory of Chemical Biology and Molecular Imaging, Graduate School of Medicine, The University of Tokyo, 7-3-1 Hongo, Bunkyo-ku, Tokyo, 113-0033 Japan; Division of Endoscopy, Hokkaido University Hospital, N14, W5, Kita-ku, Sapporo, 060-8648 Japan; Department of Otolaryngology-Head and Neck Surgery, Hokkaido University Graduate School of Medicine, N15, W7, Kita-ku, Sapporo, 060-8638 Japan; Japan Agency for Medical Research and Development (AMED)-CREST, 7-1 Ootemachi-1, Chiyoda-ku, Tokyo, 100-0004 Japan

**Keywords:** Fluorescent imaging, γ-Glutamyltranspeptidase, Head and neck squamous cell carcinoma

## Abstract

**Background:**

Detecting superficial head and neck squamous cell carcinoma (HNSCC) by endoscopy is challenging because of limited morphological hallmarks, and iodine cannot be applied to head and neck lesions due to severe mucosal irritation. γ-glutamyltranspeptidase (GGT), a cell surface enzyme, is overexpressed in several cancers, and it has been reported that γ-glutamyl hydroxymethyl rhodamine green (gGlu-HMRG), a fluorescent targeting agent which can be enzymatically activated and becomes fluorescent after cleavage of a GGT-specific sequence, can be activated within a few minutes after application to animal models. We investigated whether early HNSCC can be detected by applying gGlu-HMRG to clinical samples.

**Methods:**

gGlu-HMRG was applied to four HNSCC cell lines, and fluorescence was observed by fluorescence microscopy and flow cytometry. Immunohistological examination was performed in three recent cases of endoscopic submucosal dissection (ESD) to investigate GGT expression. Fluorescence imaging with gGlu-HMRG in eight clinical samples resected by ESD or surgery was performed, and fluorescence intensity of tumor and normal mucosa regions of interest (ROI) was prospectively measured.

**Results:**

All four gGlu-HMRG-applied cell lines emitted green fluorescence. Immunohistological examination demonstrated that GGT was highly expressed in HNSCC of the recent three ESD cases but barely in the normal mucosa. Fluorescence imaging showed that iodine-voiding lesions became fluorescent within a few minutes after application of gGlu-HMRG in all eight resected tumors. Tumor ROI fluorescence intensity was significantly higher than in the normal mucosa five minutes after gGlu-HMRG application.

**Conclusions:**

Fluorescence imaging with gGlu-HMRG would be useful for early detection of HNSCC.

## Background

Head and neck squamous cell carcinoma (HNSCC) is the sixth most common cancer in the world, with about 630,000 new cases diagnosed annually [1]. The prognosis of HNSCC is poor because it is typically detected at the advanced stage [2, 3]. However, patients with early-stage HNSCC, such as stage I and II, achieve a better prognosis with a 70–90 % 5-year survival rate as compared with patients with advanced HNSCC [4]. Therefore, early detection of HNSCC is imperative, particularly for high-risk patients, such as cigarette smokers and alcohol abusers; however, early detection of superficial HNSCC is very difficult because there are few morphological hallmarks to differentiate the disease [5]. Although iodine chromoendoscopy has been widely accepted for detection of early esophageal squamous cell carcinoma (SCC) [6, 7], it cannot be applied to head and neck lesions in conventional endoscopy because iodine causes severe mucosal irritation, which can result in aspiration into the airways [8, 9].

Although narrow band imaging (NBI) and autofluorescence imaging (AFI) have been used to detect early HNSCC, these modalities have not been widely accepted [10, 11]. Therefore, an efficient and reliable method to detect superficial HNSCC is required.

γ-glutamyltranspeptidase (GGT) is a cell surface enzyme involved in cellular glutathione metabolism and has been reported to be overexpressed in several human cancers, such as those of the lung, ovary, liver and bile duct [12–14]. GGT has been reported to play a role in tumor progression, invasion and drug resistance [12, 15]. It has been reported that γ-glutamyl hydroxymethyl rhodamine green (gGlu-HMRG), a fluorescent targeting agent that can be enzymatically activated, based on the fluorophore rhodamine green, which becomes fluorescent after cleavage of a GGT-specific sequence, was developed and gGlu-HMRG can be activated specifically in seconds to minutes by topical application [16]. It has also been demonstrated that gGlu-HMRG can improve endoscopic detection of colitis-associated cancer with a higher target-to-background ratio than conventional white light colonoscopy in a murine model [17]. However, whether fluorescence imaging with gGlu-HMRG can detect human HNSCC remains to be elucidated.

Accordingly, the aim of this study was to evaluate whether superficial HNSCC can be detected by application of gGlu-HMRG using fresh clinical samples obtained by endoscopic submucosal dissection (ESD) or local surgical resection.

## Methods

### Enzymatic-activatable fluorescent targeting agent

gGlu-HMRG was synthesized as previously described [18], and resuspended in 10 mM dimethylsulfoxide (DMSO, Sigma-Aldrich, St. Louis, Missouri) and stored at −80 °C. When used, gGlu-HMRG was thawed at room temperature and diluted to 1 or 100 μM using phosphate-buffered saline (PBS, Life Technologies, Carlsbad, California).

### Cell culture

HNSCC cell lines—HSC2, HSC3 and HSC4—were obtained from the American Type Culture Collection (ATCC; Manassas, Virginia), and cultured in Dulbecco’s Modified Eagle Medium (DMEM; Nacalai Tesque, Kyoto, Japan) supplemented with 10 % fetal bovine serum (FBS; Life Technologies), 100 U/mL of penicillin and 100 μg/mL of streptomycin (Wako Pure Chemical Industries, Osaka, Japan). SCC25 cells were obtained from the Japanese Collection of Research Bioresources (JCRB, Osaka, Japan) and cultured in DMEM/F-12 (Nacalai Tesque) supplemented with 10 % FBS, 100 U/mL of penicillin and 100 μg/mL of streptomycin. The culture was maintained at 37 °C in a humidified atmosphere of 95 % air and 5 % CO_2_.

### Fluorescence microscopy

Cells were cultured in 35-mm dishes; next, once the cells had been reached at around 80 % confluence, cells were washed with PBS, 1 μM of gGlu-HMRG was added and cells were incubated in the dark for 20 min at 37 °C. Fluorescence microscopy was performed using a Biorevo BZ-9000 microscope (Keyence, Osaka, Japan), equipped with the following filters: excitation wavelength, 450**–**490 nm; emission wavelength, 500**–**550 nm. Phase contrast images were also developed.

### Flow cytometry

Cultured cells were treated with 0.25 % trypsin/ethylenediaminetetraacetic acid (Life Technologies), harvested and resuspended in PBS. Cells (1 **×** 10^6^) were incubated in the dark with 1 μM of gGlu-HMRG for 20 min at 37 °C and analysed using a flow cytometer (FACSCanto II; Becton, Dickinson and Company, Franklin Lakes, New Jersey).

### Time course of fluorescence intensity in cultured cell lines

Cells (2 **×** 10^4^) were cultured on a black 96-well plate overnight and incubated with 1 μM of gGlu-HMRG with or without 10 μM of GGT inhibitor (GGsTop®, Wako Pure Chemical Industries). The time course of the fluorescence intensity was analysed using a microplate reader (505–555 nm; Tecan, Mannedorf, Switzerland).

### Patient studies

This study prospectively reviewed eight consecutive HNSCC tumors treated by ESD and local surgical resection in seven patients at the Department of Gastroenterology and Hepatology and the Department of Otolaryngology-Head and Neck Surgery of Hokkaido University Hospital between June 2014 and February 2016.

The indication of ESD or local surgical resection for HNSCC are as follows; 1) within slight invasion in the subepithelium and 2) no lymph node metastasis by computed tomography. ESD was performed using a single-channel gastrointestinal endoscope with a transparent attachment hood fitted to the tip using a needle knife (KD-10Q-1, Olympus, Tokyo, Japan) and insulation tip (IT knife, Olympus) under general anesthesia [19]. All ESD procedures were performed by an experienced endoscopist who had performed over 100 esophageal ESD procedures.

Local surgical resection was performed using a Colorado microdissection needle (Stryker, Kalamazoo, Michigan) and an electrosurgical generator (Force FX, Covidien, Dublin, Ireland) under general anesthesia.

The resected specimen was immediately extended on a black rubber and fixed with pins. Next, 100 μM of gGlu-HMRG was sprayed onto the specimen. Fluorescence imaging was performed using a handheld fluorescent imaging system (Discovery; INDEC Medical Systems, Santa Clara, California), which enables the capture of white-light images and fluorescent images with 450–490 nm blue excitation light. Fluorescence images were recorded 0, 0.5, 2, 5, 7, 9, 11 and 13 min after gGlu-HMRG administration. Subsequently, specimens were washed with PBS and observed using an endoscope (H260Z, Olympus) under a white light with 1.5 % iodine staining.

Fluorescence intensities were measured with Image J software (National Institutes of Health, Rockville, Maryland). Tumor and normal squamous mucosa regions of interest (ROI) were manually traced on each image. The ROI of the tumor was determined according to the iodine staining images. The mean fluorescence intensity of each ROI was calculated as pixel intensity values ranging from 0 to 255.

### Histopathology

Specimens were fixed in 40 g/L of formaldehyde saline, embedded in paraffin and cut into 5-μm sections. Tissue sections were stained with hematoxylin and eosin (H&E) and microscopically examined for histological type, tumor size, depth of invasion, lymphovascular invasion and resected margin by experienced pathologists, according to the World Health Organization (WHO) classification. Immunohistochemical analysis of GGT expression was performed using an anti-GGT1 antibody (dilution, 1:600; Abcam, Cambridge, UK).

### Statistical analysis

Data were expressed as means ± SEM. Parameters were compared between the groups using a paired *t*-test. Differences were considered significant at a *P* value < 0.05. All analyses were performed using GraphPad Prism version 6 (GraphPad Software, San Diego, California).

## Results

### Evaluation of gGlu-HMRG in HNSCC cell lines

**Fig. 1:**
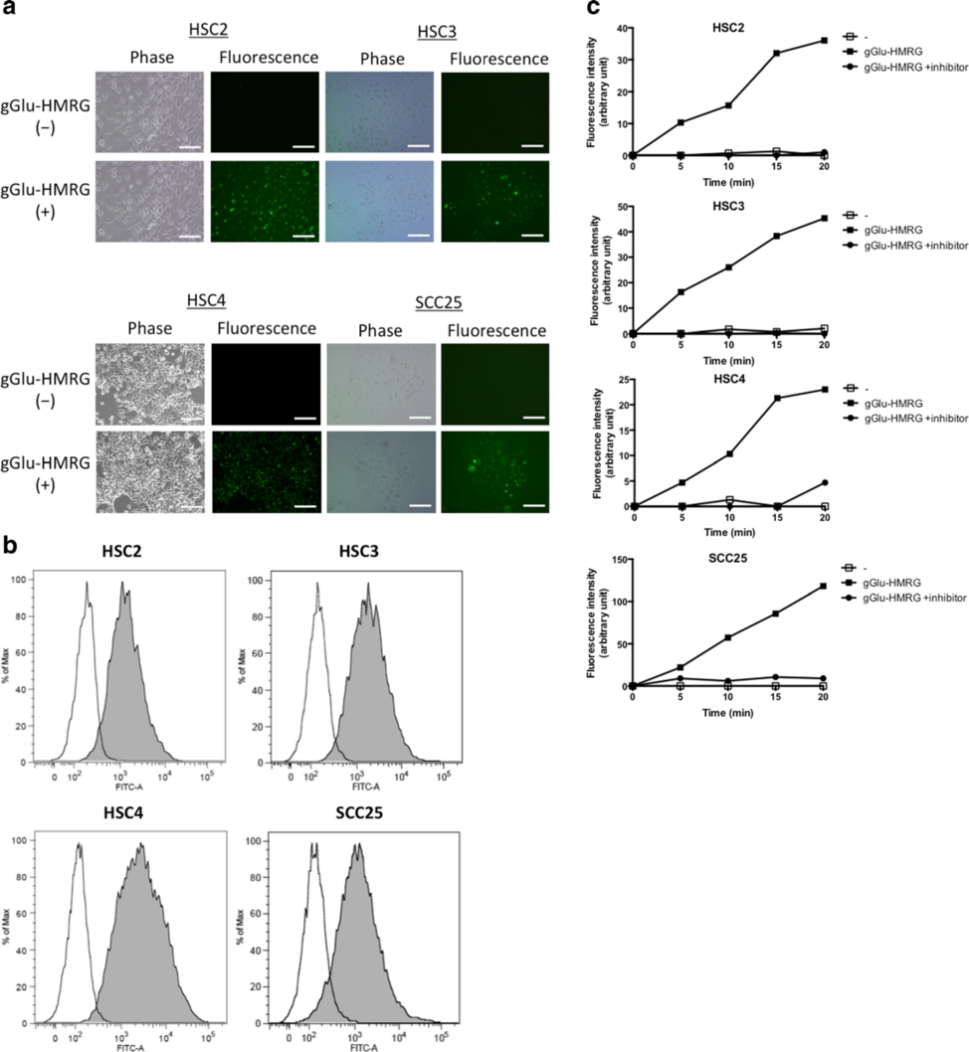
Fluorescent imaging of HNSCC cell lines *in vitro*. **a** gGlu-HMRG fluorescence was detected by fluorescence microscopy. Phase contrast images (left column), gGlu-HMRG fluorescence images (right column), Scale bars, 100 μm. **b** Flow cytometric analysis of GGT expression. Open area; no gGlu-HMRG, Closed area; with gGlu-HMRG. **c** GGT inhibition in cell lines shows decreasing GGT activity over time, resulting in low fluorescence intensity

To investigate GGT expression in HNSCC cells, gGlu-HMRG fluorescence was examined using four cell lines of HNSCC (HSC2, HSC3, HSC4 and SCC25). All tumor cell lines emitted fluorescence following gGlu-HMRG administration as evidenced by fluorescence microscopy (Fig. 1a) and flow cytometry (Fig. 1b). Fluorescence intensity was increased over time in all cell lines; however, when cultured with a GGT inhibitor, fluorescence emission was completely blocked (Fig. 1c). These results suggest that GGT is expressed in HNSCC cell lines and that gGlu-HMRG is activated by GGT.

### Expression of GGT in recent HNSCC cases treated with ESD

**Fig. 2:**
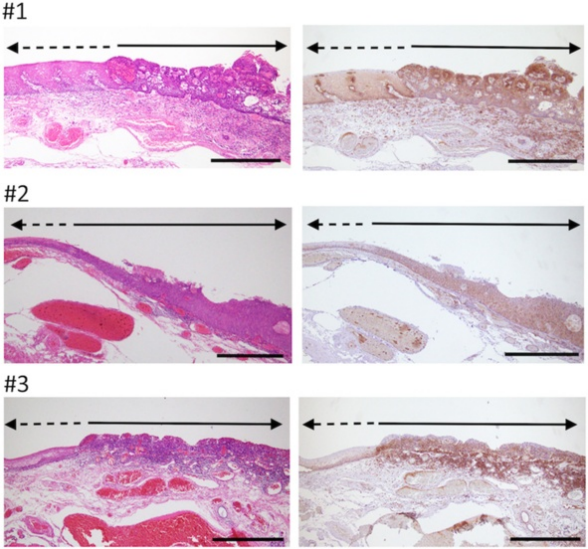
Immunohistological anaylsis of GGT expression in the past three HNSCC cases treated with ESD. Resected specimens were stained with hematoxylin and eosin (*left*) and an anti-GGT1 antibody (*right*). Scale bars, 500 μm. Dotted and solid lines indicate the part of normal eithelium and tumor, respectively

To confirm tumor-specific expression of GGT in HNSCC, we performed immunohistological examination of the tumors of three recent cases that had been treated with ESD. As shown in Fig. 2, GGT was expressed specifically in the tumor and barely expressed in the basal layer of the normal counterpart in all cases examined.

### *Ex vivo* fluorescent imaging with gGlu-HMRG of HNSCC cases treated with ESD or local surgical resection

**Fig. 3:**
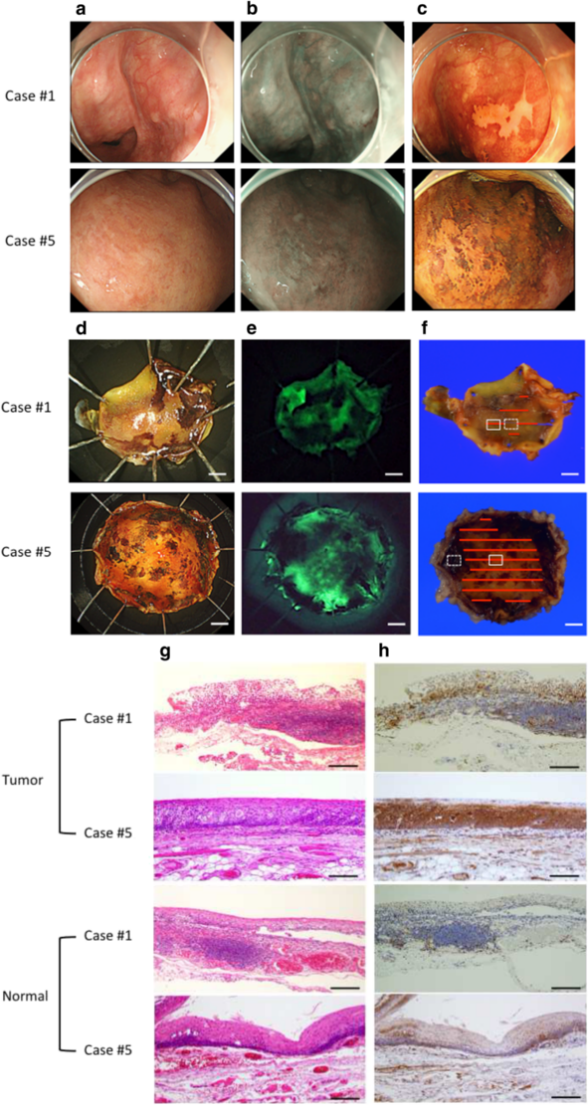
*Ex vivo* fluorescent imaging with gGlu-HMRG of two representative HNSCC cases (cases #1 and #5). **a** Endoscopic imaging with white light. **b** Narrow band imaging (NBI). **c** Iodine staining performed under general anesthesia. **d** Resected specimen observed with iodine staining. **e** Fluorescent imaging after spraying gGlu-HMRG. **f** Resected specimen mapping for tumor region. SCC was shown in red line. **g** Hematoxylin and eosin staining of the tumor and normal component. **h** Immunohistochemical examination investigating GGT expression in the tumor and normal component. Square lines in **f** correspond to the upper figures in **g** and **h**. Dotted square lines in **f** correspond to the lower figures in **g** and **h**. Scale bars of **d**-**f**, 1 mm (case#1) and 5 mm (case#5). Scale bars of **g** and **h**, 200 μm

We next evaluated whether early HNSCC can be detected by spraying gGlu-HMRG using dissected specimens. ESD and local surgical resection were performed in seven patients with eight lesions. (Table 1). It was difficult to detect the superficial tumors with white light (Fig. 3a), and all cases were barely detected using narrow band imaging (NBI, Fig. 3b). Iodine staining was performed both before resection under general anesthesia (Fig. 3c) and after resection (Fig. 3d). Resected specimens were also sprayed with gGlu-HMRG and fluorescence images were obtained (Fig. 3e). In all cases, tumor lesions became fluorescent within a few minutes corresponding to an area almost exactly identical to the iodine-unstained lesion. In several cases, the subsites of the resected mucosa became fluorescent even before applying gGlu-HMRG, and immunohistological analysis did not show any positive staining for GGT in the subsites of the resected mucosa. Therefore, we speculate that autofluorescence was emitted by the burning effect [20]. Histological analysis confirmed that the iodine-unstained and fluorescent lesion were early SCC expressing GGT in all cases (Fig. 3f–h).

**Table 1:** Patient characteristics

Patient No.	Site of lesion	Size (mm)	Treatment	Morphology	Depth
1	Hypopharynx	5 × 4	ESD	IIb	Tis
2	Hypopharynx	10 × 7	ESD	IIa + IIb	Tis
3	Hypopharynx	32 × 19	ESD	IIb + Is	T2
4	Epiglottis	12 × 9	ESD	IIb	T1
5	Soft palate	20 × 15	Surgery	IIb	T1
6	Hypopharynx	17 × 13	ESD	IIb	T1
6	Hypopharynx	7 × 6	ESD	IIb	T1
7	Hypopharynx	16 × 15	ESD	IIb + IIa	T1

*ESD* endoscopic submucosal dissection. Age ranges from 65 to 79

### Fluorescence intensity of tumor and normal epithelium after spraying gGlu-HMRG

**Fig. 4:**
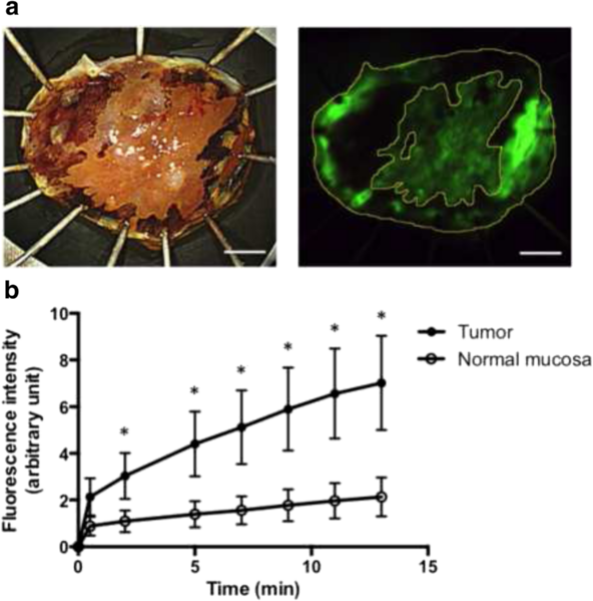
Fluorescence intensity of tumor versus normal epithelium after spraying gGlu-HMRG. **a** An example of tracing the region of interest (ROI, case #7). Scale bars, 2 mm. **b** Time course of fluorescence intensity of tumor and normal epithelium after spraying gGlu-HMRG of all eight cases

We finally measured the fluorescence intensity of both tumor and normal epithelium of all eight cases for 13 min. The tumor lesion was traced according to the iodine staining (Fig. 4a). The fluorescence intensity of the tumor lesion increased immediately after gGlu-HMRG spraying and rose to a mean intensity of 7 at 13 min, while that of normal mucosa remained <2 (Fig. 4b). The matched rate between iodine-unstained and gGlu-HMRG-induced fluorescent area was 74 %.

## Discussion

In this study, we evaluated the use of gGlu-HMRG for the detection of early HNSCC and found that (1) all HNSCC cell lines examined emitted fluorescence following gGlu-HMRG exposure; (2) HNSCC, but not normal tissue, expressed GGT in three recent ESD cases; and (3) tumor lesions became fluorescent immediately after gGlu-HMRG being applied to all eight HNSCC cases.

It is difficult to detect superficial HNSCC using conventional endoscopy with white light because mucosal changes are usually very subtle [5, 21]. Recently, NBI and AFI have been used to detect early cancers of the upper gastrointestinal tract and have been reported to be superior to white-light endoscopy in terms of sensitivity, specificity and accuracy for the diagnosis of HNSCC and esophageal SCC [10, 11, 21–26]. However, these modalities were reported from a limited number of institutes and hospitals and require remarkable expert skills to be successfully employed. In addition, it has been reported that the detection rate of early esophageal SCC using NBI was 10–13 % for SCC high-risk groups [24, 25], suggesting that it requires a sharp learning curve for the detection of HNSCC with NBI [27]. Furthermore, inflammatory changes in the larynx have been reported to cause false positive results in AFI [23]. Therefore, the development of a novel method with which even a non-expert gastroenterologist or otolaryngologist can detect early HNSCC is warranted.

When the gGlu-HMRG as targeting agent encounters GGT on the surface of cancer cells, it is hydrolysed by GGT, becoming highly fluorescent HMRG. HMRG is immediately taken up by cancer cell lysosomes through the cell membrane [16]. Therefore, HMRG is expected to emit strong fluorescence in cancer lesions. Accordingly, it has been demonstrated that topical spraying of gGlu-HMRG could provide immediate and specific enhancement of cells overexpressing GGT in animal models [16, 17]. In addition, it has been recently demonstrated, in a pilot study of fluorescence imaging of endoscopically resected colorectal tumors, that topical spraying of gGlu-HMRG enabled rapid and selective fluorescence imaging of 54 % and 76 % of adenomas and carcinomas in adenoma, respectively [28]. Although the authors used 50 or 500 μM of gGlu-HMRG, our results suggested that using 100 μM of gGlu-HMRG is sufficient for tumor detection.

Immunohistological examination demonstrated that GGT was highly expressed in tumor tissue but barely expressed at the basal lamina of the normal epithelium. The fluorescence intensity of the normal epithelium was very weak; however, it gradually became stronger with time. This is probably because it took a longer time for gGlu-HMRG to reach the basal lamina, which only weakly expresses GGT, than to reach the tumor cells.

There are several limitations to this study. Because the study was performed *ex vivo*, GGT activity may have decreased to some extent following tumor resection. It took 10–20 min to initiate fluorescence imaging after tumor resection, and imaging was performed at room temperature rather than at 37 °C. Therefore, it is expected that gGlu-HMRG would react faster in an in vivo clinical study than in an ex vivo study. In addition, because this is a pilot study investigating only eight cases, it remains to be elucidated whether all superficial HNSCC and precancer lesion can be detected with gGlu-HMRG. Future clinical trials studying a larger number of HNSCC cases would clarify these concerns.

## Conclusions

In conclusion, topical spraying of gGlu-HMRG enabled rapid and specific fluorescence imaging of superficial HNSCC, and appears to be useful in the early detection of HNSCC.

## Abbreviations

AFI, autofluorescence imaging; DMEM, Dulbecco’s Modified Eagle Medium; DMSO, dimethylsulfoxide; ESD, endoscopic submucosal dissection; FBS, fetal bovine serum; GGT, γ-glutamyltranspeptidase; gGlu-HMRG, γ-glutamyl hydroxymethyl rhodamine green; HNSCC, head and neck squamous cell carcinoma; NBI, narrow band imaging; PBS, phosphate-buffered saline; ROI, regions of interest; SCC, squamous cell carcinoma
